# Effects of Increasing Levels of Palm Kernel Oil in the Feed of Finishing Lambs

**DOI:** 10.3390/ani12040427

**Published:** 2022-02-11

**Authors:** Daniela Pionorio Vilaronga Castro, Paulo Roberto Silveira Pimentel, Jarbas Miguel da Silva Júnior, Gercino Ferreira Virgínio Júnior, Ederson Américo de Andrade, Analívia Martins Barbosa, Elzânia Sales Pereira, Claudio Vaz Di Mambro Ribeiro, Leilson Rocha Bezerra, Ronaldo Lopes Oliveira

**Affiliations:** 1 Department of Animal Science, Federal University of Bahia, Salvador 40170110, BA, Brazil; danipionorio_zootecnia@hotmail.com (D.P.V.C.); paulorobertopimentel@zootecnista.com.br (P.R.S.P.); miguelreges@gmail.com (J.M.d.S.J.); gercino.ferreiravj@yahoo.com.br (G.F.V.J.); edersonzootec@gmail.com (E.A.d.A.); analiviabarbosa@gmail.com (A.M.B.); claudioribeiro@ufba.br (C.V.D.M.R.); 2 Department of Animal Science, Federal University of Ceara, Fortaleza 60021970, CE, Brazil; elzania@hotmail.com; 3 Department of Animal Science, Federal University of Campina Grande, Patos 58708110, PB, Brazil; leilson@ufpi.edu.br

**Keywords:** crude protein, lauric acid, ruminal protozoa

## Abstract

**Simple Summary:**

Palm kernel oil (PKO) is extracted from an oleaginous seed fruit (*Elaeis guineenses* Jacq.) commonly cultivated in Brazil and can be used strategically as a ruminal fermentation modulator to improve animal performance. We conducted three experimental trials by increasing PKO levels in the diets of lambs. Although we observed low consumption of most nutrients, we also observed that feed conversion improved as the PKO inclusion level increased, indicating that the animals needed to consume less food to gain 1.0 kg of body weight. In addition, we observed that nutrient digestibility was not affected by the inclusion levels of PKO. We also did not observe differences in ruminal fermentation parameters but noted a reduction in the protozoan population. Therefore, we conclude that the inclusion of palm kernel oil may be beneficial to lambs and can lower the cost of feed in regions that contain an abundance of this byproduct.

**Abstract:**

The aim of this study was to evaluate the effects of the inclusion of palm kernel oil (PKO) in a lamb diet on nutrient intake, digestibility, ingestive behavior, nitrogen balance, blood metabolites, rumen fermentation parameters, and animal performance. Three experimental trials were conducted. The treatments consisted of varying levels of PKO included in the diet, with PKO_zero_ = no PKO inclusion, PKO_1.3_ = 1.3% addition, PKO_2.6_ = 2.6% addition, PKO_3.9_ = 3.9% addition, and PKO_5.2_ = 5.2% addition, based on the total dry matter (DM) of the diet. With the inclusion of PKO in the diet, linear decreases in DM (*p* < 0.001), crude ash (*p* < 0.001), crude protein (CP) (*p* < 0.001), neutral detergent fiber (NDF) (*p* < 0.001), nonfibrous carbohydrate (NFC) (*p* < 0.001), and total digestible nutrient (TDN) (*p =* 0.021) intake were observed, as was an increase in ether extract (EE) intake (*p* < 0.001). The digestibility coefficients of NDF and NFC were not affected by PKO addition to the diet. However, the digestibility of DM (*p =* 0.035), EE (*p* < 0.001), CP (*p* < 0.001), and TDNs (*p* < 0.001) increased when PKO was added to the lambs’ diet. Reductions in N intake (*p* < 0.001), fecal nitrogen excretion (*p* < 0.001), and microbial protein production (*p* < 0.001) were noted with increasing PKO levels. Serum cholesterol increased (*p* < 0.001) while serum GGT enzyme concentrations in the blood decreased (*p =* 0.048) with increasing PKO levels. PKO addition had no effect on total weight gain and average daily gain; however, feed conversion improved (*p =* 0.001) with increasing PKO levels. The intake, digestibility, ingestive behavior, and growth performance of lambs with PKO_1.3_ added to their diet were similar to animals that did not receive PKO, meaning that PKO could be an alternative energy source for growing lambs because it does not harm animal performance and can lower the cost of feed.

## 1. Introduction

The use of strategies to increase the efficiency of the production system promotes ruminal fermentation modulation through microbiota modification. Modulation can occur by removing groups of microorganisms, especially protozoa, which decrease the nutrients available to the animal [1]. According to Newbold and Ramos-Morales [2], the defaunation process is related to increased microbial growth efficiency due to decreased predation of bacteria by protozoa. Therefore, palm kernel oil is an interesting option for this purpose [3].

Palm kernel oil (PKO) is extracted from oleaginous seed fruits (*Elaeis guineenses* Jacq.), which are commonly found and cultivated in Brazil [4]. PKO is a source of medium-chain fatty acids, such as lauric acid, which has antimicrobial activity [5], exerting effects on bacteria [5,6,7] and protozoa [8,9]. As a result of its antiprotozoal activity, studies have been carried out to verify its effects on nutrient intake and digestibility, mainly in dairy cattle [8,9,10,11]. This inhibitory effect may be related to a lower microbial adhesion to the plant fiber caused by the lipids coating the fiber or to the possible direct cytotoxic effect of the fatty acids changing the lipid composition and physicochemical properties of the membranes, increasing the fluidity and permeability of the microbial cells [1,12,13,14]. Regarding the use of lauric acid for lambs, Machmüller and Kreuzer [15] observed no effect on the digestibility of dry matter and neutral detergent fiber when they provided increasing doses of coconut oil as a source of lauric acid to lambs; however, they identified an increase in crude protein digestibility as the concentration of lauric acid increased, and such effects were related to a decrease in the protozoan population.

The added PKO associated with corn oil in dairy cow diets increased milk production without decreasing intake or diet digestibility [3]. However, no information on palm kernel oil administration and its effects on lambs is available. Other studies using palm kernel cake have shown some benefits from its inclusion in lamb feed [16,17], which may indicate a positive impact of this oil when added to the diet. Thus, the aim of this study was to evaluate the effects of different levels of palm kernel oil in the diet of lambs, evaluating intake, performance, nutrient digestibility, ingestive behavior, and ruminal and blood parameters. Furthermore, we hypothesize that the inclusion of different levels may benefit nutrient digestibility and decrease the ruminal protozoan population.

## 2. Materials and Methods

### 2.1. Experimental Diets and Animals

The study was carried out at the experimental facilities of the School of Veterinary Medicine and Animal Science of the Federal University of Bahia (UFBA) in São Gonçalo dos Campos.

A total of 70 animals were included in three experimental trials. In all trials, the same experimental diets were used. The experimental diets were based on the inclusion of palm kernel oil (PKO; PalmFry, Jundiaí, Brazil) in the diet, with PKO_zero_ = no palm kernel oil addition, PKO_1.3_ = 1.3% palm kernel oil addition, PKO_2.6_ = 2.6% palm kernel oil addition, PKO_3.9_ = 3.9% palm kernel oil addition, and PKO_5.2_ = 5.2% palm kernel oil addition, and on the dry matter of the total diet.

The animals in all experimental trials were fed twice a day (08:00 and 16:00) a total mixed ration (TMR) diet, with feeding design ensuring 10–20% refusals, and water was provided ad libitum. Tifton-85 hay (*Cynodon* sp.) chopped into particles of approximately 5 cm was included in the diets at a 40:60 roughage:concentrate ratio. The diet compositions are described in Table 1. The diets were isonitrogenous and were formulated according to the NRC [18] to provide an average daily gain of 200 g.

### 2.2. Trial 1—Nutrient Intake, Blood Metabolites, Fatty Acids, and Chemical Diet Analysis

Nutrient intake was evaluated in 40 noncastrated male Santa Inês lambs (body weight (BW) 25.73 ± 4.01 kg), which had been previously vaccinated and dewormed and were randomly assigned to groups according to an entirely randomized design, housed in individual stalls measuring 1.0 m^2^, and suspended in wooden slats with individual water and feed troughs, with a total of eight animals per treatment.

The evaluation period was 96 d, and the first 15 d were intended for adaptation to the environment, management, and diets. The animals were weighed at the beginning of the experimental period and every 25 d to follow weight gain. Every day in the morning before feeding, the orts were collected and weighed to determine intake, allowing adjustments in the quantities to be offered. Every three days, a sample was collected for further chemical and bromatological analysis. At the end of the experimental period, the animals were weighed after a 16 h fast on solids and water to determine total weight gain (TWG), average daily gain (ADG), and the feed conversion ratio (FCR: the ratio between total feed intake and total weight gain).

Blood samples (i.e., 10 mL) were collected by jugular venipuncture, on the last day of the experiment, using a vacutainer system in tubes free of anticoagulants. After collection, the blood samples were centrifuged (Centrilab^®^ model CE3001, São Paulo, Brazil) at 5000× *g* for 20 min to obtain the serum, which was placed in Eppendorf tubes and stored in a freezer (−20 °C) for further analysis. The blood parameters were analyzed using specific commercial enzymatic kits from Labtest^®^ Diagnostica S.A. (Lagoa Santa, Minas Gerais, Brazil) using a semiautomatic biochemical analyzer (BioPlus 2000^®^, São Paulo, Brazil): albumin was measured with bromocresol green (Ref. 19); total cholesterol was measured by the cholesterol enzymatic method (Ref. 13); triglycerides were measured with glycerol phosphate oxidase (GOD; Ref. 87); the total protein concentration was measured by the biuret method (Ref. 99); and alanine aminotransferase (ALT; Ref. 108), aspartate aminotransferase (AST; Ref. 109) and gamma-glutamyltransferase (GGT; Ref. 105) were measured by UV IFCC kinetic assay.

The ingredient samples and refusals were predried at 55 °C for 72 h and ground in a 1 mm sieve in a Wiley mill (Tecnal, City of Piracicaba, State of São Paulo, Brazil) for further laboratory analyses of dry matter (DM; AOAC method 930. 15), crude protein (CP; AOAC method 968.06), ether extract (EE; AOAC method 954.05), and crude ash (AOAC method 942.05). The neutral detergent fiber (NDF) and acid detergent fiber (ADF) contents were evaluated according to Van Soest et al. [19]. The NDF content was corrected for ash and protein (NDF_ap_) using thermostable alpha-amylase without sodium sulfite, and for residual ash [20] and residual nitrogenous compounds [21]. Nonfiber carbohydrate (NFC) contents were calculated as described by Weiss [22].

The fatty acid compositions of the experimental diets were determined according to Palmquist and Jenkins [23]. A total of 0.5 g of dry sample was weighed in duplicate into Pyrex-type test tubes with a Teflon cap. In each tube, 2 mL of hexane and 3 mL of 10% methanolic acetyl chloride were added. The tubes were capped, vortexed (Fisatom 772, São Paulo, Brazil), heated in a water bath at 90 °C for 2 h, and then cooled. A total of 1 mL of hexane and 10 mL of 6% K_2_CO_3_ were added. Samples were again homogenized by vortexing. The tubes were centrifuged (Centribio 80–2B, Equipar Ltda., Paraná, Brazil) for 5 min, and the solvent layer was collected and transferred to another test tube containing 1.0 g of Na_2_SO_4_ and activated charcoal. The tubes were centrifuged, and the supernatant was transferred to small vials for gas chromatography (GC).

Fatty acid methyl esters (FAMEs) were separated in a GC (Perkin Elmer Clarus 680) equipped with a flame ionization detector (GC–FID) and an ELITE-WAX fused silica capillary column (30 m × 0.32 mm × 0.25 μm). The analysis parameters were as follows: the injector temperature was 250 °C; the detector temperature was 280 °C; and the column temperature was programmed at 150 °C for 16 min, with increases of 2 °C per minute up to 180 °C and maintenance of this temperature for 25 min, followed by increases of 5 °C up to 210 °C, which was maintained for 25 min. Helium gas was used as a carrier gas with a flow of 1 mL/min. The hydrogen gas flow rate was 30 mL/minute, and the synthetic air flow rate was 300 mL/min. Injections were performed in duplicate for each extraction, and the injection volume was 1 μL. FA was identified by comparing the retention times of the sample peaks with the retention time of the mixed standard FA (189–19, Sigma, St Louis, MO, USA). The results were quantified by area normalization and expressed in g/100 g of FAME.

### 2.3. Trial 2—Nutrient Digestibility, Nitrogen Balance and Ingestive Behavior

For the second trial, 25 noncastrated male Santa Inês lambs (34.61 ± 2.61 kg BW) were distributed according to an entirely randomized design with five treatments and five repetitions. All animals had been previously vaccinated and dewormed and were housed in individual metabolic cages (1.5 m × 0.75 m) with individual feed and water troughs, each of which had a system that allowed separate feces and urine collection.

The digestibility evaluation was performed for 21 d, including 14 d for adaptation to the facilities and experimental diets and 7 d for collection of feces, urine, feed, and orts. The feed and ort samples were analyzed as previously described. Feces were collected in individual plates and weighed before the morning feeding. After total collection, the feces were weighed and a 20% portion was separated and dried in an oven with forced air circulation at 55 °C for 72 h and subsequently analyzed.

The total digestible nutrients were calculated according to NRC [24] using the digestible fractions of nonfiber carbohydrates, crude protein, ether extract, and neutral detergent fiber. The nutrient digestibility coefficient (CD) was determined according to the equation:

CD = ((kg of ingested portion − kg of excreted portion)/(kg of ingested portion)) × 100(1)

The nitrogen balance and microbial protein production were evaluated by total urine collection. Total urine collection was carried out in individual bowls containing 50 mL of 20% sulfuric acid solution. The volume and pH were measured daily, and 10% of the total volume was stored to compose a pooled sample at the end of the collection period.

A 10 mL aliquot of the pooled urine sample was diluted in 40 mL of 0.036 N sulfuric acid (H_2_SO_4_) solution and frozen at −20 °C for further analysis of nitrogen compound excretion and purine derivatives, allantoin, uric acid, xanthine, and hypoxanthine to calculate the efficiency of microbial protein synthesis and the intestinal flow of nitrogen compounds, respectively. The nitrogen balance was determined by the difference between the amount ingested and the losses recorded in urine and feces.

The total urinary nitrogen content was determined according to the Kjeldhal method [25], and the nitrogen balance (NB) was obtained using the following equations and expressed in g/day and in g/kg^0.75^/day:

NB = N_ingested_ − (N_feces_ + N_urine_) (2)

N_absorbed_ = N_ingested_ − N_feces_ (3)

N_ingested_ = N_offered_ − N_orts_ (4)

Allantoin, xanthine, hypoxanthine, and uric acid were determined according to Chen and Gomes [26] by calculating microbial purines absorbed from the excretion of purine derivatives in urine. The intestinal flux of microbial nitrogenous compounds (N) was calculated as a function of microbial purines absorbed [26].

The ingestive behavior of the animals was evaluated during a 24 h period with observations every five minutes according to Martin and Bateson [27] by evaluating each animal individually in relation to the time spent on rumination, feeding, and idle activities. Chewing parameters were evaluated in each animal individually [28] in the morning (10:00), afternoon (15:00) and evening (21:00) by observing the number of chewing movements per bolus feeding, which was performed by previously trained evaluators using digital chronometers and artificial lighting at night, with previous adaptation of the animals. Feeding and rumination efficiencies based on DM and NDF were obtained by calculations described by Bürger et al. [29].

### 2.4. Trial 3—Ruminal Fermentation Parameters Evaluation

The ruminal fermentation parameters were evaluated using five Santa Inês sheep (52.1 ± 13.2 kg BW), cannulated in the rumen and housed individually in suspended stalls with feed and water troughs distributed in 5 × 5 Latin squares. The experiment had a duration of 55 days divided into periods of 11 days, with 10 days for adaptation to the diets and 1 day for data collection. Ruminal fluid samples (i.e., 100 mL) were collected in the morning at 0 (before feeding), 2, 4 and 6 h after feeding, and the pH of the ruminal fluid was measured immediately after collection using a digital potentiometer.

To determine the ammoniacal nitrogen (N-NH_3_) concentration, samples of approximately 25 mL of rumen fluid were filtered through cheesecloth, placed in a recipient containing 1 mL of 1:1 sulfuric acid solution and stored at −10 °C for later analysis. After thawing, the samples were distilled with 2 N KOH solution, following the procedures of Detmann et al. [25] for total nitrogen determination. A 50 mL aliquot of ruminal fluid was mixed (1:1, *v*/*v*) with a 50% formalin solution for subsequent counting of the total number of protozoa, according to the method described by Dehority and Tirabasso [30].

### 2.5. Statistical Analysis

The variables of nutrient intake and digestibility, nitrogen balance, and ingestive behavior were analyzed by means of PROC MIXED SAS^®^ 9.4 software in an entirely randomized design, using the following mathematical model:

Y_i_ = μ + T_i_ + ε_i_ (5)

where µ is the overall mean, T_i_ is the treatment effect, which is the fixed effect, and ε_i_ is the experimental error.

Heterogeneity of variances was tested by the REPEATED command and used when significant; however, the variance was not homogeneous and was therefore not considered in the final data analysis. The covariance structures “diagonal, autoregressive, unstructured, and variance compounds” were tested and defined according to the lowest value obtained for the corrected Akaike information criterion corrected (cAIC). Polynomial contrasts were used to test the linear and quadratic effects of palm kernel oil supply in the lambs’ diet on all parameters evaluated, selecting the contrast that best represents the data by the lowest value of the root mean square error (RMSE). Cubic effects were not used because there is no biological interpretation for these parameters. Initial body weight was tested as a covariate and used when significant.

For the variables of ruminal fermentation parameters (third trial), PROC MIXED was used for a Latin square design considering period and animal as random effects according to the following mathematical models; the first model was used for protozoa counts and the second for variables analyzed over time, such as pH and the ammoniacal nitrogen concentration in ruminal fluid:

Y_ijk_ = μ + D_i_ + a_j_ + p_k_ + ε_ijk_ (6)

Y_ijkl_ = μ + D_i_ + a_j_ + p_k_ + (D × a × p) + T_l_ + (D × T) + ε_ijkl_ (7)

where µ corresponds to the overall mean, D_i_ is the diet, which is a fixed effect, a_j_ corresponds to the animal, p_k_ is the period effect, which was considered a random effect, along with the animal effect; T_l_ corresponds to time, with was a fixed effect; and ε_ijkl_ is the experimental error.

The pH and ammoniacal nitrogen variables were evaluated as repeated measures over time using the cAIC value with the REPEATED command to choose the best error matrix structure. Degrees of freedom were adjusted according to Kenward–Roger by evaluating the effect of time using orthogonal contrasts.

The PROC NLIN command was used to analyze the observed linear plateau response for variables related to nitrogen balance using the lowest RMSE value among linear, quadratic, and linear plateau responses as the selection criterion. In addition, the mean of variables analysed were compared by Tukey’s test (SAS^®^ 9.4). A significant effect was declared when *p* ≤ 0.05, and a trend was declared when 0.05 < *p* ≤ 0.10.

## 3. Results

Reductions in DM (*p* < 0.001), ash (*p* < 0.001), CP (*p* < 0.001), NDF (*p* < 0.001), NFC (*p* < 0.001), and TDN (*p* < 0.001) intake with increasing PKO in the lambs’ diet were observed (Table 2). Furthermore, an effect on body and metabolic weights was identified, with reduced intakes of DM (*p* < 0.001), CP (*p* < 0.001), NDF (*p* < 0.001), and TDN (*p* < 0.001) as the level of PKO inclusion increased. EE intake in g/day (*p* < 0.001), BW (*p =* 0.029), and metabolic weight (*p =* 0.007) showed a quadratic effect, with higher intake levels of EE and higher BW when the inclusion level of PKO was 2.47%. The effectively ingested CP was not affected by the presence of PKO in the lambs’ diet. A tendency for a linear reduction in effectively ingested NDF with increasing levels of palm kernel oil inclusion was identified. PKO addition to the diet caused reductions in the effective ingestion of ash (*p* = 0.020), NFC (*p* = 0.002), and TDN (*p* < 0.001) (Table 2). The effectively ingested EE showed a quadratic effect, with higher consumption as the inclusion level of PKO increased. The digestibility of NDF was not influenced by the presence of PKO in the diet of finishing lambs. A trend toward a quadratic effect of PKO on the digestibility coefficient of NFC (*p* = 0.071) was found (Table 2). Linear increases in the digestibility coefficients of DM (*p* = 0.035), CP (*p* < 0.001), and TDN (*p* < 0.001) were also noted. A quadratic effect of the addition of PKO on the digestibility coefficient of the EE was further identified, with higher digestibility when the inclusion level was 5.2% PKO.

From the means test, the intake of lambs (g/d; g/kg BW; g/kg BW^0.75^), along with DM, ash, CP, NDF_ap_ NFC, and TDN intake, were higher (*p* < 0.05) with 0% and 1.3% of PKO inclusion (as DM total dietary) when compared to the other inclusion levels evaluated. In contrast, the control treatment (0%) promoted a lower (*p* < 0.05) EE intake (g/d; g/kg BW; g/kg BW^0.75^) in lamb, followed 1.3%; 2.6% PKO. On other hand, the inclusion of 3.9 and 5.2% of PKO had the highest EE intake. With regard to nutrient digestibility, the inclusion of 0% presented lower CP digestibility compared to other treatments.

The final weight (*p* = 0.061), total weight gain (*p* = 0.058) and average daily gain (*p* = 0.057) showed a linear decreasing trend as the level of PKO in the diet increased, as well as a linear decreasing effect for feed conversion, with better feed conversion for animals fed diets containing the highest level of PKO (*p* = 0.001; Table 3). Also, 3.9 PKO inclusion was associated with lower (*p* < 0.05) total weight gain and ADG compared to other treatments, while the inclusion of 5.2% PKO was associated with the best feed conversion (*p* < 0.05).

PKO addition to the diet reduced the N retained by lambs until the inclusion level of 2.62% of this fatty acid in the lambs’ diet (∀ x ≤ 2.62 Y= 6.380; ∀ x > 2.62 Y = 6.38 + 1.7546 × (2.6272 − X); RMSE = 3.963), with stabilization in its values as the concentration of PKO increased until the inclusion level of 5.2% (Table 4). No effect of PKO on the microbial protein production efficiency of lambs was found. As the level of PKO supply in the diet increased, reductions in N intake (*p* < 0.001), fecal N excretion (*p* < 0.001), and microbial protein production (*p* < 0.001) occurred. There was a quadratic effect for urinary N excretion (*p* = 0.048), with higher urinary N excretion when the inclusion level of PKO in the diet was 5.2%. In means test comparisons, N intake and microbial protein production (g/day) was similarly high for 0 (control), 1.3%, and 2.6 PKO inclusion (as DM total dietary) compared to 3.9 and 5.2% PKO inclusion in the lambs’ diet, and the inclusion of 5.2% PKO was associated with a lower intake. N fecal excretion was greater (*p* < 0.05) for groups 0 (control) and 1.3% PKO inclusion compared to 2.6, 3.9%, and 5.2% PKO inclusion in the lambs’ diet, which were associated with similar N fecal excretion values.

PKO inclusion in the lambs’ diet linearly increased the serum cholesterol concentration (*p* < 0.001). In contrast, a linear decrease in gamma-glutamyltransferase (GGT) enzyme blood concentration (*p* = 0.048) due to PKO addition in the lambs’ diet was observed. Serum concentrations of total proteins (*p* = 0.860), albumins (*p* = 0.574), globulins (*p* = 0.736), and triglycerides (*p* = 0.144), and the ratio between albumins and globulins (*p* = 0.593), as well as the enzymatic concentrations of aspartate aminotransferase (*p* = 0.308) and alanine aminotransferase (*p* = 0.230), were not affected by increasing levels of PKO in the lambs’ diet (Table 4).

The means test demonstrated that no PKO inclusion was associated with lower (*p* < 0.05) cholesterol (mg/dL) compared to other treatments. In contrast, the inclusion of 5.2% of PKO in the lambs’ diet promoted the lower ruminal protozoa count compared to other treatments.

**Figure 1:**
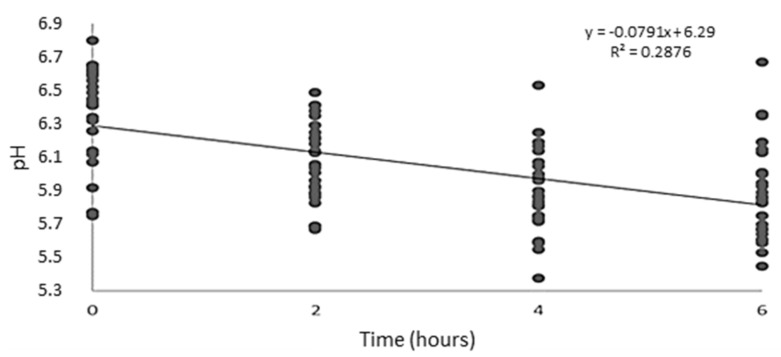
Effect of feeding time on ruminal fluid pH of lambs fed diets containing palm kernel oil.

A decrease in ruminal fluid pH values over time was noted after feed intake (*p* < 0.001; Figure 1). PKO inclusion in the lambs’ diet did not affect the pH of the ruminal fluid of lambs or the ammoniacal nitrogen concentration. However, PKO addition to the diet reduced the total protozoa count (*p* = 0.002) of the ruminal fluid of lambs linearly (Table 4).

The addition of PKO to the lambs’ diet did not affect the time spent ruminating (*p* = 0.72) or idling (*p* = 0.84; Table 5). A linear increasing trend was observed for time spent feeding (*p* = 0.078) and number of chews per bolus (*p* = 0.066). The feeding efficiency of DM (*p* = 0.008) and NDF (*p* = 0.018), as well as the rumination efficiency of DM (*p* < 0.001) and NDF (*p* < 0.001), decreased linearly with the inclusion of PKO in the lambs’ diet.

From the means test, it was observed that feed efficiency (g/h) of DM and NDF was similarly high with 0 (control), 1.3, 2.6, and 5.2% PKO inclusion (as DM total dietary) compared to 3.9 PKO inclusion in the lambs’ diet. DM and NDF rumination efficiency was similarly high with 0 (control) and 1.3% PKO inclusion compared to other treatments.

## 4. Discussion

The DM intake reduction observed in the present study consequently reduced the intake of other nutrients (except for the ether extract) and the feeding (g DM/h) and rumination (g DM/h and g NDF/h) efficiencies as PKO inclusion increased. As a result, this lower intake consequently resulted in more time spent feeding, probably due to feed selection behavior and lower animal performance. PKO addition resulted in lower nutrient intake. Allen [31] suggested that this effect on intake may be related to the impact of fat on ruminal fermentation, the acceptability of diets, the release of gut hormones, such as cholecystokinin, that act on the satiety control center, and the effect of lipid oxidation in the liver. However, despite this low intake and lower performance, the inclusion of PKO in the diet indicated better utilization of the ingested feed according to the digestibility and feed conversion data. This is probably due to the higher energy density as the inclusion of oil increased. Furthermore, it is also interesting to note that this did not affect fiber digestibility, even with reduced intake. According to Palmquist and Jenkins [32], high lipidic levels in ruminant diets (>70 g/kg DM) can inhibit ruminal fermentation, negatively affect fiber digestibility and modify the microbial population [1,33]. However, we did not observe any effect on ruminal fermentation.

The fatty acids present in PKO (Supplementary Table S1) were related to the reduction in the protozoan population [34]. In our study, we evaluated only the ruminal protozoan population and observed that PKO addition in the diet of lambs up to the level of 5.2% dry matter caused a decrease in these microorganisms without causing effects on ruminal fermentation parameters, which may be related to the presence of lauric acid, as observed by [8,15,35] and Matsumoto et al. [36], who reported the toxic effect of lauric acid on some ruminal microorganisms, especially protozoa [1]. Indeed, this effect can be explained by the fact lauric acid, a medium-chain fatty acid with characteristics similar to polyunsaturated fatty acids, can be adsorbed to the microbial surface and incorporated into the membrane, promoting changes in its permeability and fluidicity [13,14,37], leading to membrane destabilization and potentially impairing microbial development. This corroborates our data, since we also observed lower microbial protein synthesis with the inclusion of palm kernel oil. However, the reduction of the protozoan population may be beneficial, since its presence in the ruminal environment is closely related to energy loss in the form of methane [38,39].

Despite the effect on microbial protein production, the efficiency of microbial protein production was not affected by the presence of this source of fatty acids in the diet of animals. The efficiency of microbial protein production is closely related to the amount of microbial protein that reaches the small intestine, and the increase in the nitrogenous compound concentration in serum and urine indicates a reduction in production efficiency [1,40]. Thus, even with reduced intake and decreased microbial protein production, microbial protein production efficiency remained unaffected due to increased nitrogen availability and decreased nitrogen losses in the ruminal environment. Under conditions of low N intake, more recycling of metabolized nitrogen in the form of urea occurs, which may lead to a decrease in N excretion due to higher utilization by the animal [41,42], as verified in the present study.

The inclusion of PKO affected only two blood metabolites evaluated. The plasma concentration of cholesterol increased with the increased inclusion of palm kernel oil in the diet. According to Mayes and Botham [43], the presence of saturated fatty acid sources, such as PKO, in the diet promotes the formation of smaller VLDL particles, which are used by the extrahepatic tissues more slowly when compared to larger particles, and the decrease in absorption rates by the tissues could increase circulating cholesterol, causing an increase in serum concentrations of this component. The GGT serum concentration was reduced by the presence of palm kernel oil, and this enzyme is normally associated with liver status, being particularly linked to long-term liver injury [44]. Most likely, this increased dietary lipid content can have positive effects on the liver of lambs; however, all the mean values observed are within the range considered normal [45,46].

## 5. Conclusions

The inclusion of 1.3% palm kernel oil (PKO) in a lamb diet was associated with intake, digestibility, ingestive behavior, and growth performance in lambs similar to animals that did not receive PKO. In contrast, the inclusion of 5.2% of PKO in total DM dietary reduced intake, despite increasing digestibility and feed conversion and reducing fecal nitrogen excretion. Thus, PKO, up to 1.3% DM total, can be an alternative energy source for growing lambs. It is important to note that the use of this byproduct is recommended when it is easily available and cost-effective.

## Figures and Tables

**Table 1:** Ingredient proportions, chemical composition, and fatty acid profile of the experimental diets.

	Palm Kernel Oil Levels (% DM Total)				
	0	1.3	2.6	3.9	5.2
**Ingredients (% Total Diet)**					
Hay	40.0	40.0	40.0	40.0	40.0
Ground corn	42.5	41.0	39.6	38.1	36.6
Soybean meal	16.0	16.2	16.3	16.5	16.7
Palm kernel oil	0.0	1.30	2.60	3.90	5.19
Mineral mixture ^1^	1.50	1.50	1.50	1.50	1.50
**Chemical Composition (% Dry Matter)**					
Dry matter (% fresh matter)	86.8	86.9	87.1	87.3	87.5
Organic matter	93.9	93.9	93.9	94.0	94.0
Crude ash	6.1	6.1	6.1	6.0	6.0
Crude protein	13.9	13.9	13.8	13.8	13.7
Ether extract	1.40	2.66	3.92	5.18	6.44
Neutral Detergent Fiber_ap_	36.6	36.5	36.4	36.3	36.2
Acid Detergent Fiber	17.1	17.1	17.1	17.0	17.0
Non-Fibrous Carbohydrates ^2^	42.0	40.1	39.8	38.7	37.6
**Fatty Acid Profile (g/100 g Total Fat)**					
C 10:0 (Capric)	0.38	2.00	2.36	2.58	2.78
C 12:0 (Lauric)	3.92	28.20	33.94	36.46	38.61
C 14:0 (Myristic)	2.84	10.62	11.77	13.00	14.20
C 16:0 (Palmitic)	56.00	52.70	49.05	50.70	50.80
C 16:1 (Palmitoleic)	0.42	0.34	0.35	0.34	0.32
C 18:0 (Stearic)	9.80	9.74	9.02	7.35	9.56
C 18:1^cis9^ (Oleic)	42.17	32.67	31.17	29.27	27.57
C 18:2^cis9 cis12^ (Linoleic)	54.60	33.10	31.90	28.60	24.70
C 18:3n-3 (Linolenic)	11.18	10.41	10.40	10.38	10.07
Others	6.28	9.52	9.74	11.38	11.67

^1^ Assurance levels (per kilogram of active elements): 120 g of calcium, 87 g of phosphorus, 147 g of sodium, 18 g of sulfur, 590 mg of copper, 40 mg of cobalt, 20 mg of chromium, 1800 mg of iron, 80 mg of iodine; 1300 mg of manganese, 15 mg of selenium; 3800 mg of zinc, 300 mg of molybdenum; maximum 870 mg of fluoride. ^2^ NFC = 100 − NDF_ap_ − CP − EE.

**Table 2:** Nutrient intake (g/day; g/kg BW; g/kg BW^0.75^) and digestibility coefficient of finishing lambs fed diets containing palm kernel oil (PKO).

Item	Palm Kernel Oil Levels (%DM Dietary Total)					SEM ^1^	*p*-Value ^2^		
	0	1.3	2.6	3.9	5.2		L	Q	Effect
**Nutrient Intake (g/day)**									
Dry matter	1113.0 ^a^	1092.3 ^a^	861.6 ^b^	816.9 ^b^	673.1 ^c^	37.6	<0.01	0.68	<0.01
Ash	68.0 ^a^	65.9 ^a^	50.9 ^b^	48.2 ^b^	39.7 ^c^	2.31	<0.01	0.94	<0.01
Crude protein	166.2 ^a^	161.1 ^a^	129.6 ^b^	115.2 ^bc^	97.9 ^c^	5.43	<0.01	0.73	<0.01
Ether extract	17.1 ^d^	32.1 ^c^	38.7 ^b^	49.4 ^a^	54.4 ^a^	1.14	<0.01	<0.01	<0.01
NDF_ap_^3^	345.4 ^a^	344.1 ^a^	258.1 ^b^	261.0 ^b^	187.5 ^c^	16.8	<0.01	0.39	<0.01
NFC ^4^	516.3 ^a^	489.2 ^a^	384.3 ^b^	343.1 ^bc^	293.3 ^c^	14.5	<0.01	0.74	<0.01
TDN ^5^	755.5 ^a^	808.9 ^a^	654.7 ^b^	623.7 ^bc^	616.7 ^c^	35.6	0.02	0.99	0.02
**Nutrient Intake as Body Weight (g/kg BW)**									
Dry matter	34.0 ^a^	31.9 ^a^	27.2 ^b^	25.8 ^b^	20.5 ^c^	0.93	<0.01	0.32	<0.01
Crude protein	5.09 ^a^	4.72 ^a^	4.10 ^b^	3.65 ^b^	2.93 ^c^	0.14	<0.01	0.37	<0.01
Ether extract	0.52 ^e^	0.94 ^d^	1.21 ^c^	1.55 ^b^	1.76 ^a^	0.04	<0.01	0.03	<0.01
NDF_ap_	10.5 ^a^	10.0 ^ab^	8.09 ^bc^	8.23 ^c^	5.75 ^d^	0.46	<0.01	0.18	<0.01
NFC	15.8 ^a^	14.3 ^b^	12.2 ^c^	10.9 ^c^	8.89 ^d^	0.37	<0.01	0.95	<0.01
TDN	20.9 ^a^	21.7 ^a^	17.3 ^b^	16.8 ^b^	14.5 ^c^	1.28	<0.01	0.65	<0.01
**Nutrient Intake by Body Weight (g/kg BW^0.75^)**									
Dry matter	81.3 ^a^	77.2 ^a^	64.4 ^b^	61.2 ^b^	48.9 ^c^	2.29	<0.01	0.40	<0.01
Crude Protein	12.1 ^a^	11.4 ^a^	9.71 ^b^	8.64 ^b^	7.03 ^c^	0.34	<0.01	0.45	<0.01
Ether Extract	1.24 ^e^	2.26 ^d^	2.88 ^c^	3.68 ^b^	4.16 ^a^	0.08	<0.01	<0.01	<0.01
NDF_ap_^3^	25.1 ^a^	24.3 ^a^	19.3 ^b^	19.6 ^b^	13.5 ^c^	1.12	<0.01	0.22	<0.01
NFC ^4^	37.8 ^a^	34.6 ^a^	28.8 ^b^	25.8 ^b^	21.3 ^c^	0.89	<0.01	0.96	<0.01
TDN ^5^	51.3 ^a^	53.6 ^a^	42.9 ^b^	41.5 ^b^	36.0 ^c^	3.18	<0.01	0.60	<0.01
**Effective Nutrient Intake (g/100g)**									
Ash	6.11	6.03	5.92	5.89	5.88	0.08	0.02	0.52	0.17
Crude protein	14.9	14.8	15.1	14.1	14.4	0.38	0.20	0.93	0.36
Ether extract	1.53 ^e^	2.94 ^d^	4.48 ^c^	6.02 ^b^	8.53 ^a^	0.09	<0.01	<0.01	<0.01
NDF_ap_^3^	30.8	31.4	29.8	31.9	27.2	1.22	0.09	0.16	0.06
NFC ^4^	46.6 ^a^	44.8 ^ab^	44.6 ^ab^	42.0 ^b^	44.0 ^ab^	0.78	<0.01	0.14	<0.01
TDN ^5^	67.3 ^c^	71.7 ^bc^	76.1 ^ab^	79.1 ^ab^	78.7 ^a^	1.68	<0.01	0.10	<0.01
**Nutrient Digestibility Coefficient (%)**									
Dry matter	68.9	72.3	74.5	75.5	74.1	1.78	0.04	0.13	0.28
Crude Protein	69.8 ^b^	74.3 ^ab^	77.6 ^a^	79.6 ^a^	78.3 ^a^	1.60	<0.01	0.05	0.30
Ether Extract	63.4 ^d^	81.2 ^c^	88.4 ^b^	94.1 ^a^	94.2 ^a^	1.12	<0.01	<0.01	<0.01
NDF_ap_^3^	40.6	47.2	47.1	51.7	42.2	4.03	0.21	0.26	0.45
NFC ^4^	88.8	89.8	90.4	89.7	88.0	0.73	0.65	0.07	0.33
TDN ^5^	67.3 ^c^	71.7 ^bc^	76.1 ^ab^	79.1 ^ab^	76.7 ^a^	1.67	<0.01	0.10	<0.01

^1^ Standard error of the mean. ^2^ Significance at *p* < 0.05 and trend between *p* ≤ 0.05 and *p* ≤ 0.10; L, linear; Q, quadratic. ^3^ Neutral detergent fiber corrected for ash and protein. ^4^ Nonfibrous carbohydrates. ^5^ Total digestible nutrients. ^a–e^ Means followed by different letters differ by Tukey’s test (*p* < 0.05).

**Table 3:** Performance of finishing lambs fed diets containing palm kernel oil (PKO).

Item	Palm Kernel Oil Levels (%DM Dietary Total)					SEM ^1^	*p*-Value ^2^		
	0	1.3	2.6	3.9	5.2		L	Q	Effect
Initial body weight (kg)	25.10	26.85	26.40	26.48	23.83	-	-	-	
Final body weight (kg)	40.12	42.68	37.94	37.44	39.02	1.22	0.06	0.63	0.13
Total weight gain (kg)	14.40 ^ab^	16.92 ^a^	12.20 ^ab^	11.70 ^b^	13.32 ^ab^	1.19	0.06	0.59	0.03
Average daily gain (kg)	0.18 ^ab^	0.21 ^a^	0.15 ^ab^	0.14 ^b^	0.16 ^ab^	0.01	0.06	0.59	0.03
Feed conversion (kg/kg)	6.91 ^a^	5.39 ^ab^	6.40 ^a^	5.73 ^ab^	4.05 ^b^	0.51	<0.01	0.45	0.02

^1^ Standard error of the mean. ^2^ Significance at *p* < 0.05 and trend between *p* ≤ 0.05 and *p* ≤ 0.10; L, linear; Q, quadratic. ^a,b^ Means followed by different letters differ by Tukey’s test (*p* < 0.05).

**Table 4:** Nitrogen balance, production and efficiency of microbial protein production, and blood and ruminal parameters of finishing lambs fed diets containing palm kernel oil (PKO).

Item	Palm Kernel Oil Levels (%DM Dietary Total)					SEM ^1^	*p*-Value ^2^		
	0	1.3	2.6	3.9	5.2		L	Q	Effect
**Nitrogen Balance (g/day)**									
N intake	26.8 ^a^	27.0 ^a^	21.4 ^ab^	18.3 ^b^	18.2 ^b^	1.10	<0.01	0.73	<0.01
N fecal	8.01 ^a^	6.93 ^a^	4.83 ^b^	3.73 ^b^	3.67 ^b^	0.45	<0.01	0.09	<0.01
N urinary	8.55	9.99	10.9	8.46	7.64	0.99	0.30	0.04	0.18
N retained	10.3	10.1	5.72	6.07	6.77	1.86	0.08	0.37	0.24
**Microbial Protein (g/day)**									
Production	38.0 ^a^	33.5 ^ab^	28.1 ^abc^	23.9 ^bc^	19.8 ^c^	2.83	<0.01	0.56	<0.01
Efficiency	45.5	41.5	43.3	38.6	37.2	3.97	0.14	0.93	0.23
**Blood Parameters**									
Proteins (g/dL)	7.60	7.59	7.91	7.48	7.61	0.16	0.86	0.96	0.92
Albumins (mg/dL)	3.43	3.91	3.45	3.37	3.53	0.18	0.57	0.71	0.28
Globulins (mg/dL)	4.17	3.68	4.24	4.11	4.08	0.24	0.74	0.79	0.51
A:G Ratio ^3^	0.83	1.14	0.91	0.84	0.88	0.12	0.59	0.40	0.35
Cholesterol (mg/dL)	53.7 ^b^	72.7 ^ab^	91.5 ^a^	80.7 ^ab^	91.5 ^a^	7.18	<0.01	0.10	<0.01
Triglycerides (mg/dL)	15.2	13.2	15.7	17.4	17.9	2.02	0.14	0.58	0.49
GGT ^4^ (UI/L)	49.2	52.2	44.7	45.5	44.4	2.49	0.04	0.99	0.15
AST ^5^ (UI/L)	79.0	66.2	84.2	85.3	76.2	7.52	0.31	0.47	0.09
ALT ^6^ (UI/L)	12.4	11.5	15.3	14.8	14.4	2.07	0.23	0.23	0.31
**Ruminal Parameters**									
pH	6.10	5.99	5.93	6.08	6.16	0.11	0.57	0.16	0.46
N-NH_3_	18.7	17.5	19.0	17.1	19.2	1.89	0.85	0.75	0.93
Protozoa (×10^6^ mL^−1^)	8.87 ^b^	14.3 ^a^	4.52 ^c^	3.00 ^d^	0.72 ^e^	1.47	<0.01	0.38	<0.01

^1 ^Standard error of the mean. ^2^ Significance at *p* < 0.05 and trend between *p* ≤ 0.05 and *p* ≤ 0.10; L, linear; Q, quadratic. ^3^ Albumin: Globulin ratio. ^4^ Gamma-glutamyl transferase enzyme. ^5^ Aspartate-aminotransferase enzyme; ^6^ Alanine-aminotransferase enzyme. ^a–e^ Means followed by different letters differ by Tukey’s test (*p* < 0.05).

**Table 5:** Ingestive behavior of finishing lambs fed diets containing palm kernel oil (PKO).

**Item**	**Palm Kernel Oil Levels (%DM Dietary Total)**					**SEM ^1^**	***p*-Value ^2^**		
	**0**	**1.3**	**2.6**	**3.9**	**5.2**		**L**	**Q**	**Effect**
**Intake (g/d)**									
Dry matter	1117.1 ^a^	1127.9 ^a^	863.5 ^ab^	789.5 ^b^	692.4 ^b^	65.5	<0.01	0.92	<0.01
NDF	324.9 ^a^	371.1 ^a^	259.5 ^ab^	254.4 ^ab^	209.5 ^b^	23.5	<0.01	0.40	<0.01
**Ingestive Behavior (min/day)**									
Feed	208	183	219	270	230	21.7	0.08	0.86	0.19
Rumination	475	461	533	483	446	31.8	0.72	0.17	0.40
Idleness	757	796	688	687	798	42.2	0.84	0.13	0.22
**Chewing (Frequency or Number of Events)**									
No./bolus	55.2	50.0	57.7	65.3	66.5	2.81	0.07	0.59	0.31
Seg/bolus	41.4	38.9	46.1	47.9	43.8	1.69	0.28	0.56	0.43
**Feed Efficiency (g/h)**									
Dry matter	337.8 ^ab^	338.3 ^a^	237.1 ^ab^	177.9 ^b^	247.8 ^ab^	37.0	<0.01	0.20	0.03
NDF	112.0 ^ab^	137.9 ^a^	71.1 ^ab^	57.2 ^b^	73.4 ^ab^	14.3	0.02	0.60	0.02
**Rumination Efficiency (g/h)**									
Dry matter	141.7 ^a^	148.2 ^a^	98.3 ^b^	99.8 ^b^	92.6 ^b^	8.57	<0.01	0.46	<0.01
NDF	45.4 ^a^	48.8 ^a^	29.2 ^b^	32.1 ^b^	27.7 ^b^	2.83	<0.01	0.54	<0.01

^1^ Standard error of the mean. ^2^ Significance at *p* < 0.05 and trend between *p* ≤ 0.05 and *p* ≤0.10; L, linear; Q, quadratic. ^a,b^ Means followed by different letters differ by Tukey’s test (*p* < 0.05).

## Data Availability

Data are not publicly available due to restrictions on the research group but can be requested from the corresponding author.

## Supplementary Materials

The following are available online at https://www.mdpi.com/article/10.3390/ani12040427/s1, Table S1: Fatty acid composition of palm kernel oil.
