# Supplementary material for: Effects of Increasing Levels of Palm Kernel Oil in the Feed of Finishing Lambs
Source: Animals (Basel). 2022 Feb 11;12(4):427. doi: 10.3390/ani12040427 (PMC8868089; doi:10.3390/ani12040427)
Supplement: Supplementary file 1 [file animals-12-00427-s001.zip › animals-1489729-supplementary.pdf]

Supplementary

Table S1. Fatty acid composition of palm kernel oil.

Fatty acid	Systematic Name	Common Name	g/100g
C4:0 to C10:0	-	-	7,78
C12:0	Dodecanoic	Lauric	46,63
C14:0	Tetradecanoic	Myristic	16,05
C16:0	Hexadecanoic	Palmitic	8,59
C18:0	Octadecanoic	Stearic	2,34
$C18:1^{cis9}$	Octadecaenoic	Oleic	14,06
Others	-	-	4,53
Total	-	-	100,0
